# Supplementary material for: GhENODL6 Isoforms from the Phytocyanin Gene Family Regulated Verticillium Wilt Resistance in Cotton
Source: Int J Mol Sci. 2022 Mar 8;23(6):2913. doi: 10.3390/ijms23062913 (PMC8955391; doi:10.3390/ijms23062913)
Supplement: Supplementary file 1 [file ijms-23-02913-s001.zip › ijms-1606084-supplementary.pdf]

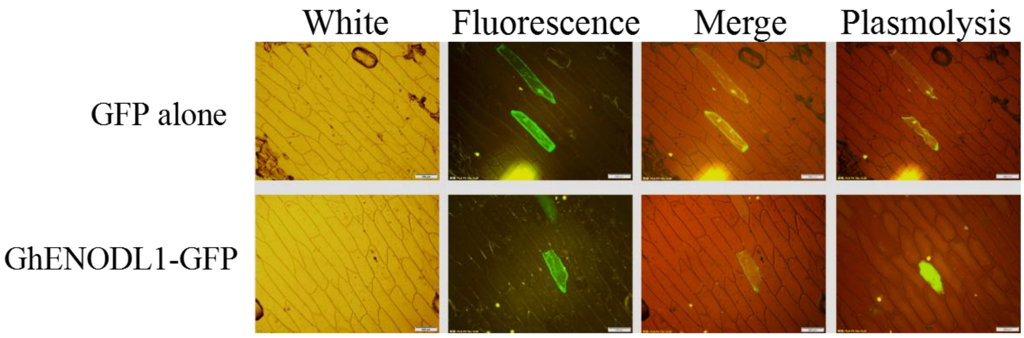

Supplemental files 1:Figure S1

Subcellular localization of GFP alone or GhENODL1-GFP fusion on onion epidermis transiently transformed by Particle bombardment. The green fluorescence were monitored using a confocal laser scanning microscope. Bars = 200 μm.

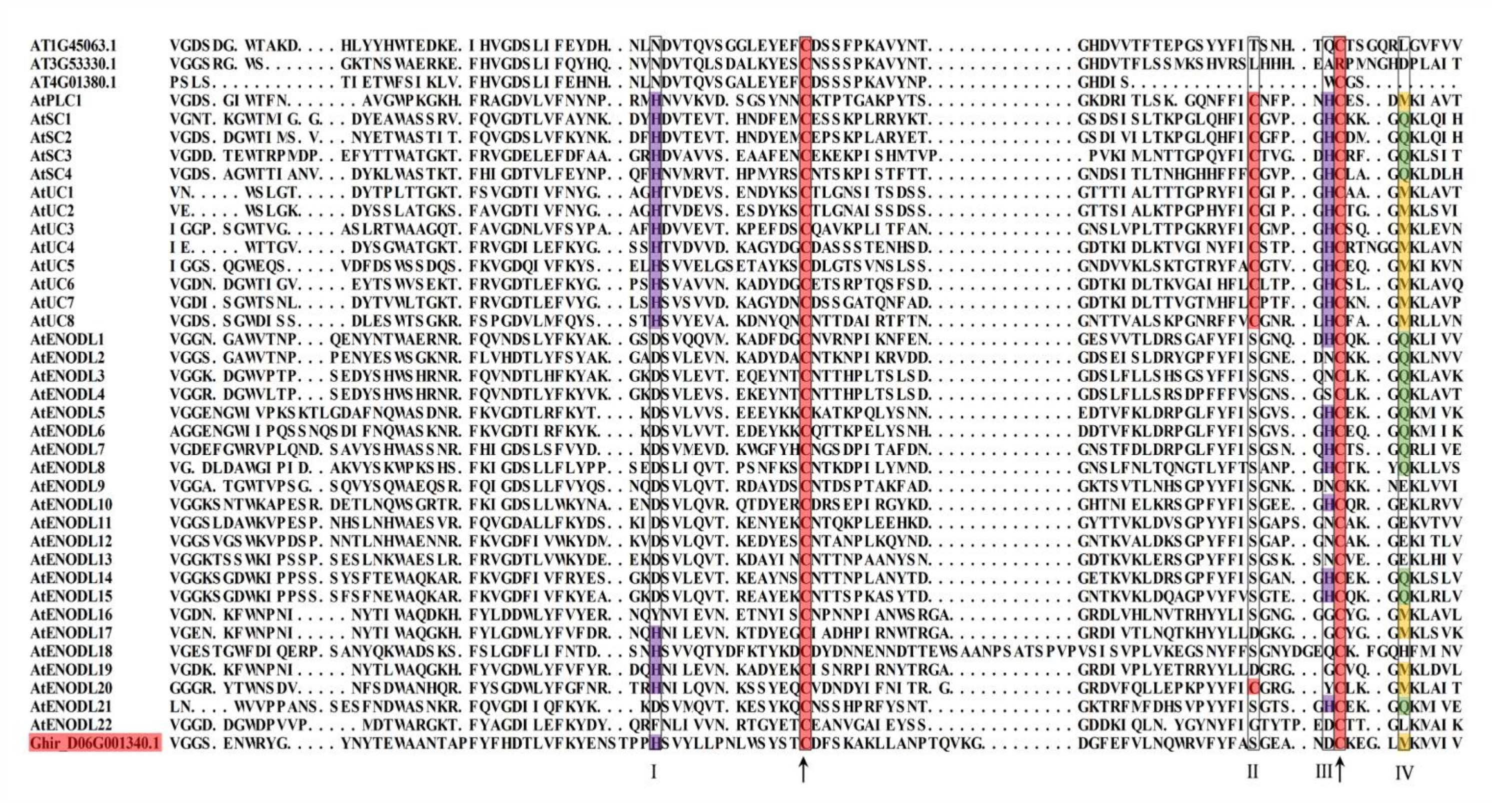

Supplemental files 2:Figure S2

Multiple Alignment of the Amino Acid Sequences of the PCNL Domains of the *A. thaliana* Phytocyanin Family and target gene *GhENODL1*(Ghir_D06G001340.1).The Cys residues involved in the disulfifide linkage are indicated in red and the amino acid residues (His, Cys, His and Gln/Met) involved in Cu binding are indicated in Ⅰ Ⅱ Ⅲ Ⅳ respectively.

Supplemental files 3: Table S1

PCs Identifified in *G. hirsutum* Genomes

Name	Gene ID	Length (aa)	MW (kDa)	Isoelectric Point	Charge
GhPC1	Ghir_D06G011310	107	11.763	10.232	7
GhPC2	Ghir_A06G007340	127	13.789	5.518	-2
GhPC3	Ghir_D11G003980	127	14.585	6.33	-0.5
GhPC4	Ghir_D05G005610	128	14.513	10.255	13
GhPC5	Ghir_A11G004040	137	15.901	7.546	3
GhPC6	Ghir_D06G001340	139	15.707	7.403	1
GhPC7	Ghir_A06G001480	139	15.717	7.243	0.5
GhPC8	Ghir_A05G010410	154	17.903	9.444	6.5
GhPC9	Ghir_A05G006200	159	17.165	9.592	6.5
GhPC10	Ghir_D07G003740	161	17.383	9.387	5
GhPC11	Ghir_A05G013540	167	18.494	6.778	1
GhPC12	Ghir_A13G003590	167	18.904	10.573	19
GhPC13	Ghir_D11G007700	167	18.685	9.529	7
GhPC14	Ghir_D04G004910	170	19.555	6.783	0.5
GhPC15	Ghir_A05G038140	171	19.764	6.941	1
GhPC16	Ghir_A05G012210	172	18.63	8.313	3.5
GhPC17	Ghir_D05G011920	173	18.682	8.313	3.5
GhPC18	Ghir_A11G007760	175	19.405	9.265	6
GhPC19	Ghir_D11G007710	175	19.359	9.299	6
GhPC20	Ghir_D05G006280	176	18.939	10.309	11.5
GhPC21	Ghir_A07G003720	178	19.011	9.605	6
GhPC22	Ghir_D05G013280	180	19.862	6.618	0.5
GhPC23	Ghir_A13G003640	184	21.078	9.286	8
GhPC24	Ghir_A05G005530	185	20.409	10.016	10.5
GhPC25	Ghir_D13G025800	188	21.473	9.039	7
GhPC26	Ghir_A13G013740	194	21.579	6.673	0.5
GhPC27	Ghir_D13G014420	194	21.634	6.344	-0.5
GhPC28	Ghir_A02G010500	196	21.218	6.739	0.5
GhPC29	Ghir_D03G009570	196	21.18	8.19	3.5
GhPC30	Ghir_A13G014720	217	23.245	4.915	-3.5
GhPC31	Ghir_D13G015400	217	23.329	4.637	-5.5
GhPC32	Ghir_D07G015760	229	24.983	6.657	0.5
GhPC33	Ghir_A07G015510	231	24.864	4.829	-4.5
GhPC34	Ghir_D05G007560	234	25.916	4.128	-14.5
GhPC35	Ghir_D05G037420	242	26.017	5.545	-3
GhPC36	Ghir_A04G002000	243	26.253	5.2	-4
GhPC37	Ghir_A03G017320	268	29.84	6.492	0

GhPC38	Ghir_D02G018560	269	30.009	7.361	2.5
GhPC39	Ghir_A13G015310	338	35.113	9.043	12.5
GhPC40	Ghir_D13G016040	338	35.2	9.043	12.5
GhPC41	Ghir_A04G003340	832	88.307	7.018	17.5
GhPC42	Ghir_D04G015900	115	12.455	7.439	1.5
GhPC43	Ghir_A06G003430	127	13.698	4.426	-4
GhPC44	Ghir_D06G003450	141	15.577	4.684	-3
GhPC45	Ghir_A09G026390	158	17.061	6.488	0
GhPC46	Ghir_A05G017860	161	17.24	4.607	-4.5
GhPC47	Ghir_A04G011560	165	17.192	8.226	4.5
GhPC48	Ghir_A07G024480	167	18.025	7.383	1.5
GhPC49	Ghir_D07G024530	167	17.953	7.383	1.5
GhPC50	Ghir_D05G017860	171	18.184	4.938	-4
GhPC51	Ghir_D05G027080	171	17.992	6.214	-0.5
GhPC52	Ghir_A05G027060	172	18.222	7.857	2.5
GhPC53	Ghir_A06G003420	175	18.017	4.992	-2.5
GhPC54	Ghir_D06G003440	175	17.999	4.992	-2.5
GhPC55	Ghir_A08G006290	177	17.806	4.305	-4
GhPC56	Ghir_D08G006420	177	17.782	4.305	-4
GhPC57	Ghir_A06G016540	179	19.466	4.763	-4.5
GhPC58	Ghir_D03G011670	184	19.68	6.233	-0.5
GhPC59	Ghir_D06G017430	184	20.136	4.48	-7
GhPC60	Ghir_A03G007100	187	19.968	6.228	-0.5
GhPC61	Ghir_D05G022710	188	19.292	6.746	0.5
GhPC62	Ghir_A05G022790	192	19.586	6.738	0.5
GhPC63	Ghir_A12G014590	204	20.971	4.303	-7
GhPC64	Ghir_D12G014730	209	21.427	4.181	-8
GhPC65	Ghir_A13G005810	216	22.805	4.161	-8
GhPC66	Ghir_D13G006500	258	27.022	3.894	-15
GhPC67	Ghir_A02G007150	82	8.94	9.856	7
GhPC68	Ghir_D05G024440	94	9.932	9	4
GhPC69	Ghir_A09G009400	98	10.681	4.162	-6.5
GhPC70	Ghir_A09G009410	109	11.846	6	-1
GhPC71	Ghir_D09G009120	109	11.863	5	-2
GhPC72	Ghir_D09G009110	119	13.016	5	-5
GhPC73	Ghir_D01G018560	123	13.098	10.446	14.5
GhPC74	Ghir_A01G017010	123	13.257	10.679	16.5
GhPC75	Ghir_D04G018430	126	13.281	10	12
GhPC76	Ghir_A04G013970	127	13.255	10.02	10.5
GhPC77	Ghir_A05G024630	127	13.346	9.605	8
GhPC78	Ghir_D02G007560	339	36.175	9.082	12
GhPC79	Ghir_A08G007230	157	17.562	9	8
GhPC80	Ghir_A11G025230	161	17.416	9	5
GhPC81	Ghir_A11G025240	162	17.378	8	3

GhPC82	Ghir_D11G025370	162	17.566	8.052	2.5
GhPC83	Ghir_D11G025380	162	17.52	8.052	2.5
GhPC84	Ghir_A01G016200	164	18.134	5	-6
GhPC85	Ghir_A12G023640	166	17.721	7	2
GhPC86	Ghir_D12G023630	166	17.721	7.383	1.5
GhPC87	Ghir_A05G001590	167	17.827	5	-4
GhPC88	Ghir_D06G005780	167	17.876	5.07	-3
GhPC89	Ghir_D05G001780	169	17.973	4.392	-5.5
GhPC90	Ghir_A08G018330	170	18.606	7	2
GhPC91	Ghir_D06G009280	170	18.164	9.379	8
GhPC92	Ghir_D01G017730	172	18.94	5	-6
GhPC93	Ghir_D08G007280	176	19.406	8.097	5
GhPC94	Ghir_D05G001760	180	19.072	7.193	2.5
GhPC95	Ghir_D11G013060	187	19.621	8.63	6
GhPC96	Ghir_A09G022890	189	19.813	9	8
GhPC97	Ghir_D09G022120	189	19.853	9.166	8
GhPC98	Ghir_A05G013010	200	21.162	4	-6
GhPC99	Ghir_D05G012760	200	21.157	4.354	-5.5
GhPC100	Ghir_D13G022130	200	20.488	4.469	-4
GhPC101	Ghir_A08G007220	205	22.038	8	4
GhPC102	Ghir_A13G021270	208	21.375	5	-3
GhPC103	Ghir_A11G025250	214	23.121	7	1
GhPC104	Ghir_D11G025390	255	27.285	4.972	-2
GhPC105	Ghir_D11G025400	259	27.691	5.004	-2
GhPC106	Ghir_D13G022140	268	28.239	10.033	12
GhPC107	Ghir_A13G010380	299	31.515	8	3
GhPC108	Ghir_A13G021280	347	35.14	8	7
GhPC109	Ghir_A13G021260	429	45.418	5	-14
GhPC110	Ghir_D13G022120	556	60.623	4.82	-16.5
GhPC111	Ghir_A05G043430	111	12.338	6.925	1
GhPC112	Ghir_D05G024780	111	12.38	6.925	1
GhPC113	Ghir_D05G001770	169	18.56	4.873	-3.5

Supplemental files 3: Table S2

ENODLs Identifified in *G. hirsutum* Genomes

Name	Accession ID	Length(aa)	singleIP	GPI	N-glyco	AG	Extensin
GhENODL1	Ghir_D06G011310	107	-	+	-	+	-
GhENODL2	Ghir_A06G007340	127	+	-	+	-	-
GhENODL3	Ghir_D11G003980	127	+	-	+	-	-
GhENODL4	Ghir_D05G005610	128	+	+	+	+	-
GhENODL5	Ghir_A11G004040	137	+	-	+	-	-
GhENODL6	Ghir_D06G001340	139	+	-	+	-	-
GhENODL7	Ghir_A06G001480	139	+	-	+	-	-
GhENODL8	Ghir_A05G010410	154	-	-	+	-	-
GhENODL9	Ghir_A05G006200	159	-	+	+	+	-
GhENODL10	Ghir_D07G003740	161	+	+	+	+	-
GhENODL11	Ghir_A05G013540	167	+	+	+	+	-
GhENODL12	Ghir_A13G003590	167	+	-	+	-	-
GhENODL13	Ghir_D11G007700	167	-	+	+	-	-
GhENODL14	Ghir_D04G004910	170	+	+	+	+	-
GhENODL15	Ghir_A05G038140	171	+	+	+	+	-
GhENODL16	Ghir_A05G012210	172	+	-	+	+	-
GhENODL17	Ghir_D05G011920	173	+	-	+	+	-
GhENODL18	Ghir_A11G007760	175	+	+	+	+	-
GhENODL19	Ghir_D11G007710	175	+	+	+	+	-
GhENODL20	Ghir_D05G006280	176	-	+	+	+	-
GhENODL21	Ghir_A07G003720	178	+	+	+	+	-
GhENODL22	Ghir_D05G013280	180	+	+	+	+	-
GhENODL23	Ghir_A13G003640	184	+	-	+	-	-
GhENODL24	Ghir_A05G005530	185	+	+	+	+	-
GhENODL25	Ghir_D13G025800	188	+	-	+	-	-
GhENODL26	Ghir_A13G013740	194	+	+	+	+	+
GhENODL27	Ghir_D13G014420	194	+	+	+	+	-
GhENODL28	Ghir_A02G010500	196	+	-	+	+	-
GhENODL29	Ghir_D03G009570	196	+	-	+	+	-
GhENODL30	Ghir_A13G014720	217	+	+	+	+	+
GhENODL31	Ghir_D13G015400	217	+	+	+	+	+
GhENODL32	Ghir_D07G015760	229	+	-	+	+	+
GhENODL33	Ghir_A07G015510	231	+	-	+	+	+
GhENODL34	Ghir_D05G007560	234	+	-	+	+	-
GhENODL35	Ghir_D05G037420	242	+	-	+	+	+
GhENODL36	Ghir_A04G002000	243	+	-	+	+	+
GhENODL37	Ghir_A03G017320	268	+	+	+	+	+

GhENODL38	Ghir_D02G018560	269	+	+	+	+	+
GhENODL39	Ghir_A13G015310	338	+	+	+	+	+
GhENODL40	Ghir_D13G016040	338	+	+	+	+	+
GhENODL41	Ghir_A04G003340	832	-	-	+	+	+

Supplemental files 3:Table S3

Genes ontology (GO) analysis involved in biological processes from differentially expressed genes by comparing *GhENODL1-4* transgenic line with WT.

Category	GOID	Description	Gene Ratio	Bg Ratio	pvalue
BP	GO:0050832	defense response to fungus	134	298	4.55E-23
BP	GO:0006955	immune response	159	390	1.05E-21
BP	GO:0002376	immune system process	168	426	4.58E-21
BP	GO:0045087	innate immune response	154	382	1.70E-20
BP	GO:0009814	defense response, incompatible interaction	90	194	5.41E-17
BP	GO:0006979	response to oxidative stress	156	451	9.99E-14
BP	GO:0009627	systemic acquired resistance	41	79	2.22E-10
BP	GO:0009751	response to salicylic acid	82	219	1.09E-09
BP	GO:0000302	response to reactive oxygen species	70	179	2.10E-09
BP	GO:0031347	regulation of defense response	92	265	9.33E-09
BP	GO:0071555	cell wall organization	143	492	4.99E-07
BP	GO:0044550	secondary metabolite biosynthetic process	75	160	1.07E-14
BP	GO:0052542	defense response by callose deposition	16	23	3.36E-07
BP	GO:0070588	calcium ion transmembrane transport	30	65	1.51E-06

BP	GO:0009620	response to fungus	162	368	2.21E-26
BP	GO:0019761	glucosinolate biosynthetic process	31	53	7.03E-10
BP	GO:0009625	response to insect	21	30	3.84E-09
BP	GO:0009753	response to jasmonic acid	82	235	4.53E-08

Supplemental files 3: Table S4

Genes ontology (GO) analysis involved in biological processes from differential genes by comparing inoculated and non-inoculated *V. dahliae* in *GhENODL1-4* transgenic line.

Category	GO ID	Description	Gene Ratio	Bg Ratio	pvalue
BP	GO:0009620	response to fungus	134	360	2.54E-15
BP	GO:0006091	generation of precursor metabolites and energy	163	479	2.89E-14
BP	GO:0006979	response to oxidative stress	153	449	1.59E-13
BP	GO:0050832	defense response to fungus	106	290	7.64E-12
BP	GO:0009735	response to cytokinin	91	238	1.37E-11
BP	GO:0002376	immune system process	141	430	3.45E-11
BP	GO:0016052	carbohydrate catabolic process	95	258	5.78E-11
BP	GO:0009814	defense response, incompatible interaction	77	195	8.34E-11
BP	GO:0045087	innate immune response	126	386	5.28E-10
BP	GO:0006955	immune response	128	394	5.43E-10
BP	GO:0008652	cellular amino acid biosynthetic process	78	219	1.62E-08
BP	GO:0009751	response to salicylic acid	78	220	2.04E-08
BP	GO:0000302	response to reactive oxygen species	65	174	3.13E-08
BP	GO:0009069	serine family amino acid metabolic	33	72	3.70E-07

BP	GO:0009817	defense response to fungus, incompatible interaction	25	53	4.98E-06
BP	GO:0009414	response to water deprivation	136	373	1.05E-14
BP	GO:0006520	cellular amino acid metabolic process	154	457	3.70E-13
BP	GO:0006790	sulfur compound metabolic process	125	388	1.55E-09
BP	GO:0009627	systemic acquired resistance	31	79	3.99E-05
BP	GO:0016051	carbohydrate biosynthetic process	123	351	4.64E-12

Supplemental files 3: Table S5

Genes ontology (GO) analysis involved in biological processes from differential genes by comparing inoculated and non-inoculated *V. dahliae* in WT.

Category	GOID	Description	Gene Ratio	Bg Ratio	pvalue
BP	GO:0009991	response to extracellular stimulus	38	258	5.04E-14
BP	GO:0031667	response to nutrient levels	34	221	3.07E-13
BP	GO:0042594	response to starvation	30	176	5.10E-13
BP	GO:0009267	cellular response to starvation	23	143	8.85E-10
BP	GO:0071456	cellular response to hypoxia	10	28	1.83E-08
BP	GO:0042737	drug catabolic process	26	210	2.17E-08
BP	GO:0016036	cellular response to phosphate starvation	14	69	8.91E-08
BP	GO:0098754	detoxification	25	213	1.16E-07
BP	GO:0009636	response to toxic substance	32	334	2.51E-07
BP	GO:0009409	response to cold	36	408	3.46E-07
BP	GO:0050832	defense response to fungus	28	309	4.18E-06
BP	GO:0009631	cold acclimation	10	51	8.65E-06
BP	GO:0009414	response to water deprivation	31	377	9.51E-06
BP	GO:0000272	polysaccharide catabolic process	18	158	1.03E-05
BP	GO:0046677	response to antibiotic	28	330	1.44E-05
BP	GO:0009415	response to water	31	385	1.44E-05

Supplemental files 3: Table S6

Primers used in this study

Primer names	Sequence(5'to3')	Purpose
GhENODL1-V-F	GAATTCATGGCTTCAATTGGTTTC	VIGS vector
GhENODL1-V-R	CGGTACCTTAAAAACGTGGCC	VIGS vector
GhENODL1-F	CGATGGCTTCAATTGGTTTCA	cloning of GhENODL1
GhENODL1-R	TTAAAAACGTGGCCAAGGGA	cloning of GhENODL1
GhENODL1-RT-F	CCTCTTGGTTGCTTTAGGTGCTT	qRT-PCR
GhENODL1-RT-R	CGCCCATTCGGTGTAATTGTAG	qRT-PCR
GhENODL1-G-F	GGGGACAAGTTTGTACAAAAAAGCAG GCTTCATGGCTTCAATTGGTTTCA	Gateway vector
GhENODL1-G-R	GGGGACCACTTTGTACAAGAAAGCTGG GTGAAAACGTGGCCAAGGGA	Gateway vector
AtEF-1α-F	AACGGTGCCAGTGGGACG	qRT-PCR
AtEF-1α-R	CCTTGACAGCAACATTCTTGACAT	qRT-PCR
AtEDS1-F1	CTGTGGAAATGGCTGTGAGGAGT	qRT-PCR
AtEDS1-R1	CGGCAAGTGCGTGACTGAAGATA	qRT-PCR
AtNDR1-F1	TCTTATGGCTTAGTCTCCGTGCG	qRT-PCR
AtNDR1-R1	CGAGGTGGACATCGTCGTAGTAG	qRT-PCR
AtPDF1.2-Q-F	CTTGTTCTCTTTGCTGCTTTC	qRT-PCR
AtPDF1.2-Q-R	CATGTTTGGCTCCTTCAAG	qRT-PCR
AtPR1-Q-F	GCTACGCAGAACAACTAAG	qRT-PCR
AtPR1-Q-R	CCAGACAAGTCACCGCTACC	qRT-PCR
AtPR2-Q-F	GTCTCAAGGAAGGTTCAG	qRT-PCR
AtPR2-Q-R	CGTTATCAACAGTGGACT	qRT-PCR
AtPR3-Q-F	CGGCGACCTCCTTCTTCCTC	qRT-PCR
AtPR3-Q-R	GAATGATGCCGCTTGTCCTG	qRT-PCR
AtPR5F	ACTTCACTCTAAGGAACAAT	qRT-PCR
AtPR5R	GTTACACATCTACCGTTTC	qRT-PCR
AtRPP4F	ATATACATGTGAACGTTTCTG	qRT-PCR
AtRPP4R	CTATATCAACATCAGCATCATC	qRT-PCR
GhUBQ14-F	CAACGCTCCATCTTGTCCTT	qRT-PCR
GhUBQ14-R	TGATCGTCTTTCCCGTAAGC	qRT-PCR
GhEDS1-F	GTGTTTACTGGACATTCATCTGG	qRT-PCR
GhEDS1-R	CTTGCTTCTTTGTCTACCGT	qRT-PCR
GhNDR1-F	CCCGTAACCAAGGAGGCTGT	qRT-PCR
GhNDR1-R	CTGCTAAGGGAAGGCAAGGATAG	qRT-PCR
GhNPR1-F	GTCTGGCTGATGTCAATCTGCG	qRT-PCR
GhNPR1-R	TCCTTCCCTTGCTCTGTCTTGG	qRT-PCR
GhPR1F	AAGAATGTGGGTTAGTGAGAGGGT	qRT-PCR
GhPR1R	ACCACTTGAGTATAATGCCCGC	qRT-PCR
GhPR2F	CCACCAGCAGCAGAAGTTATCG	qRT-PCR

GhPR2R	TTCAAGGTTTGCACTCGGAAGA	qRT-PCR
GhPR3F	ACTCCACAATCACCGAAGCCAT	qRT-PCR
GhPR3R	GCATTCCAACCCTTACCACATTC	qRT-PCR
GhPR4F	TTGCGGCAATGGCTTCAATC	qRT-PCR
GhPR4R	TGCTCTCACATTATTCGGCA	qRT-PCR
GhPR5F	GCCGTGATTCATACAGTTATCCTCA	qRT-PCR
GhPR5R	TTGGCTCTTACTTCCGACCATCT	qRT-PCR
GhPR6F	CTGGGTGTCCTGGGAAGAAC	qRT-PCR
GhPR6R	TTGTAGGGGGACGAACAACG	qRT-PCR
GhPR9F	CAACAGCGCCAACATACAGAG	qRT-PCR
GhPR9R	CAGCACAAGAGACAATGCCAG	qRT-PCR
GhPR16F	CCCAAAGCTTGCCAAAGCA	qRT-PCR
GhPR16R	GAACATTGGCTGGAGTGACC	qRT-PCR
ITS-F	CATCAGTCTCTCTGTTTATACCAACG	qRT-PCR
ITS-R	CGATGCGAGCTGTAACTACTACGCAA	qRT-PCR
